# Does evaluation of the ligamentous compartment enhance diagnostic utility of sacroiliac joint MRI in axial spondyloarthritis?

**DOI:** 10.1186/s13075-015-0729-8

**Published:** 2015-09-13

**Authors:** Ulrich Weber, Walter P. Maksymowych, Stanley M. Chan, Kaspar Rufibach, Susanne J. Pedersen, Zheng Zhao, Veronika Zubler, Mikkel Østergaard, Robert GW Lambert

**Affiliations:** King Christian 10th Hospital for Rheumatic Diseases, Gråsten, and Sygehus, Sønderjylland, Denmark; Institute of Regional Health Research, University of Southern Denmark, Odense, Denmark; Department of Rheumatology, Balgrist University Hospital, Zurich, Switzerland; Department of Medicine, University of Alberta, Edmonton, AB Canada; Department of Ophthalmology, University of Alberta, Edmonton, Alberta Canada; Rufibach rePROstat, Biostatistical Consulting and Training, Engelgasse 123, 4052 Basel, Switzerland; Copenhagen Center for Arthritis Research Center for Rheumatology and Spine Diseases, Rigshospitalet, Glostrup, and, Department of Clinical Medicine, University of Copenhagen, Copenhagen, Denmark; Department of Rheumatology, PLA General Hospital, Beijing, China; Department of Radiology, Balgrist University Hospital, Zurich, Switzerland; Department of Radiology and Diagnostic Imaging, University of Alberta, Edmonton, Alberta Canada

## Abstract

**Introduction:**

Inflammation of the sacroiliac joints (SIJ) is a fundamental clinical feature of axial spondyloarthritis (SpA). The anatomy of the irregularly shaped SIJ is complex with an antero-inferior cartilaginous compartment containing central hyaline and peripheral fibrocartilage, and a dorso-superior ligamentous compartment. Several scoring modules to systematically assess SIJ magnetic resonance imaging (MRI) in SpA have been developed. Nearly all of them are based on the cartilaginous joint compartment alone. However, there are only limited data about the frequency of inflammatory lesions in the ligamentous compartment and their potential diagnostic utility in axial SpA. We therefore aimed to evaluate the ligamentous compartment on sacroiliac joint MRI for lesion distribution and potential incremental value towards diagnosis of SpA over and above the traditional assessment of the cartilaginous compartment alone.

**Methods:**

Two independent cohorts of 69 and 88 consecutive back pain patients ≤50 years were referred for suspected SpA (cohort A) or acute anterior uveitis plus back pain (cohort B). Patients were classified according to rheumatologist expert opinion based on clinical, radiographic and laboratory examination as having nonradiographic axial SpA (nr-axSpA; n = 51), ankylosing spondylitis (n = 34), or nonspecific back pain (NSBP; n = 72). Five blinded readers assessed SIJ MRI globally for presence/absence of SpA. Bone marrow edema (BME) and fat metaplasia were recorded in the cartilaginous and ligamentous compartment. The incremental value of evaluating the ligamentous additionally to the cartilaginous compartment alone for diagnosis of SpA was graded qualitatively. We determined the lesion distribution between the two compartments, and the impact of the ligamentous compartment evaluation on diagnostic utility.

**Results:**

MRI bone marrow lesions solely in the ligamentous compartment in the absence of lesions in the cartilaginous compartment were reported in just 0–2.0/0–4.0 % (BME/fat metaplasia) of all subjects. Additional assessment of the ligamentous compartment was regarded as essential for diagnosis in 0 and 0.6 %, and as contributory in 28.0 and 7.7 % of nr-axSpA patients in cohorts A and B, respectively. Concomitant BME in both compartments was evident in 11.6–42.0 % of nr-axSpA and 2.1–2.4 % of NSBP patients.

**Conclusion:**

Assessing the ligamentous compartment on SIJ MRI provided no incremental value for diagnosis of axial SpA. However, concomitant BME in both compartments may help discriminate nr-axSpA from NSBP.

## Introduction

Inflammation of the sacroiliac joints (SIJ) is a fundamental clinical feature of axial spondyloarthritis (SpA), and sacroiliitis on magnetic resonance imaging (MRI) represents a major criterion in the Assessment of SpondyloArthritis International Society (ASAS) classification for axial SpA [1]. The anatomy of the irregularly curved SIJ is complex with an antero-inferior cartilaginous compartment, which contains central hyaline and peripheral fibrocartilage, and a dorso-superior ligamentous compartment [2, 3]. Small case series and anecdotal reports in patients with early spondyloarthritis suggest that inflammation starts in the subchondral bone marrow of the cartilaginous SIJ compartment [4–12].

**Fig. 1:**
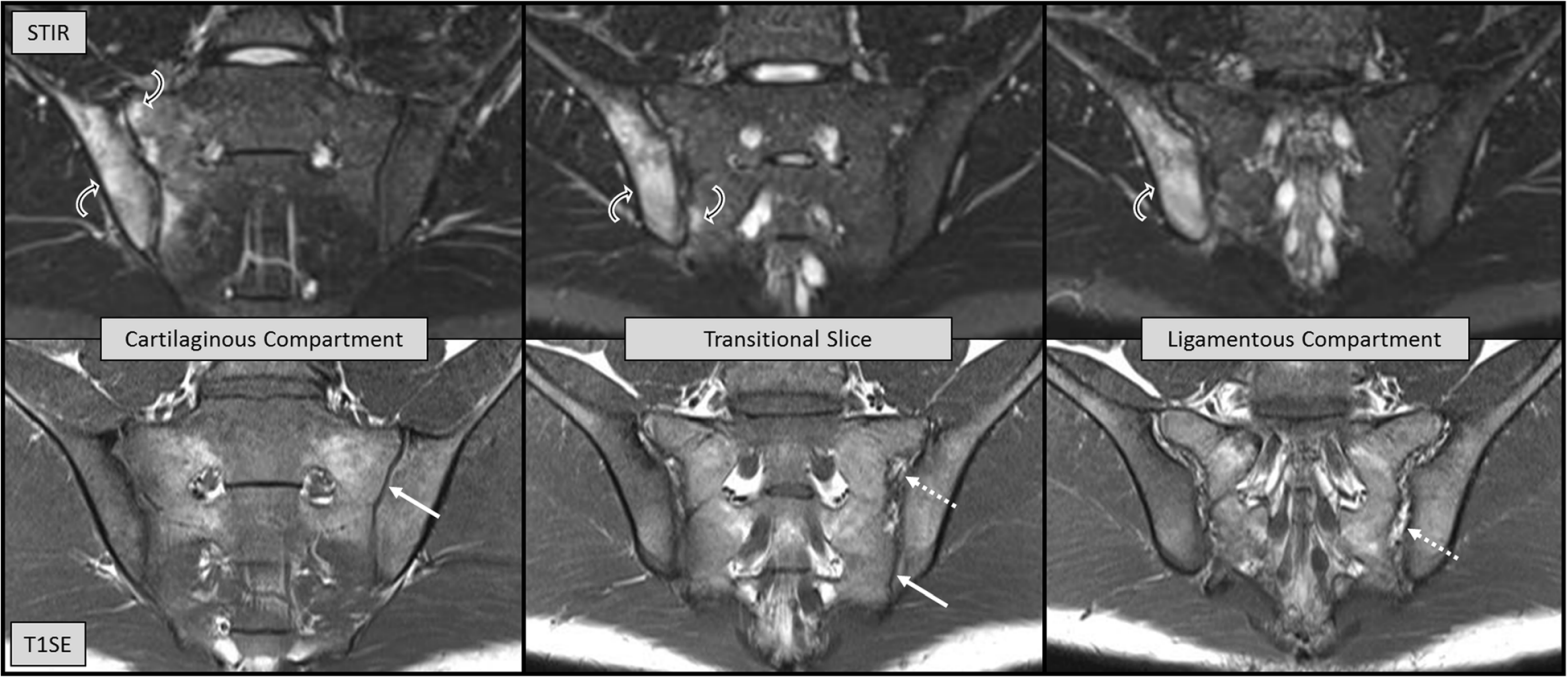
MRI studies of a 22-year-old man, human leucocyte antigen B27-positive, with a 4-month history of inflammatory back pain. The radiographic modified New York criteria were not met, and C-reactive protein was 7 mg/l (reference limit ≤5 mg/l). The upper and lower rows show short tau inversion recovery (STIR) and corresponding T1 spin echo (T1SE) sequences, respectively. The left panel represents an MRI slice from the cartilaginous compartment (*solid white arrow* on T1SE), the right panel from the ligamentous compartment (*broken white arrow* on T1SE), and the central panel is a transitional slice containing parts of both the cartilaginous and ligamentous compartment (*solid and broken white arrows* on T1SE). The black and white pattern of the space between the ilium and the sacrum in the ligamentous compartment has its origin on the T1SE images in sacroiliac ligaments which have low MRI signal intensity surrounded by fat tissue which is bright on T1SE. On the STIR sequence, the ligamentous compartment is comprised of low signal ligaments and fat tissue as the fat signal is suppressed on STIR, and the bright signal relates to fluid in the lumen of blood vessels. In this patient with nonradiographic axial spondyloarthritis, bone marrow edema (*curved white and black arrows* on STIR) predominantly in the iliac part of the right sacroiliac joint extends from the cartilaginous into the ligamentous compartment

Several scoring modules to systematically assess SIJ MRI in SpA have been developed. Nearly all of them are based on the cartilaginous joint compartment alone [13–19], and magnetic resonance images are acquired in a tilted semi-coronal plane running parallel to the posterior aspect of the sacrum. When scrolling from anterior to posterior through semi-coronal magnetic resonance images of the SIJ, the cartilaginous compartment is displayed on the first 5–6 MRI slices. The subsequent 1–2 slices have been termed “transitional” slices as they show both cartilaginous and ligamentous portions. The most posterior 3–4 slices depict the ligamentous compartment (Fig. 1). An assessment module of the SIJ developed by a Danish working group also scores inflammation in the bone marrow of the ligamentous SIJ compartment [20–22]. Magnetic resonance images for the Danish method are obtained not only in a semi-coronal but also in a semi-axial plane perpendicular to the long axis of the SIJ, which allows simultaneous assessment of the bone marrow in the ligamentous and cartilaginous compartments on the same image. However, there are only limited data about the frequency of inflammatory lesions in the ligamentous compartment and their incremental value towards diagnostic utility in axial SpA over and above the assessment of the cartilaginous compartment alone.

The goals of this study on SIJ MRI in two axial SpA inception cohorts were: (1) to systematically evaluate the frequency of bone marrow edema (BME) and fat metaplasia in the bone marrow of both the cartilaginous and ligamentous SIJ compartment by semi-coronal magnetic resonance images in SpA patients and controls; and (2) to assess the incremental value of the additional evaluation of the ligamentous compartment on diagnostic utility over and above the traditional assessment of the cartilaginous compartment alone in nonradiographic axial SpA (nr-axSpA) patients.

## Methods

### Subjects

The demographical and clinical characteristics of the study sample have been described elsewhere [23]. Two different approaches were used to recruit two inception cohorts of consecutive back pain patients aged ≤50 years newly referred to two university outpatient departments. Back pain patients referred by rheumatologists and primary care physicians for further evaluation of clinically suspected SpA were enrolled in cohort A (n = 69; University of Zurich). The same university clinic concomitantly recruited 20 age-matched healthy controls from hospital staff, defined by the Nordic questionnaire [24] and by absence of clinical features indicative for SpA. Patients of cohort B (n = 88; University of Alberta) presented with acute anterior uveitis (AAU) to a university ophthalmology department and were referred to rheumatology of the same university hospital for assessment of SpA if they indicated past or present back pain for ≥3 months on a structured questionnaire. Cohort A and B, being based on two different recruitment strategies, reflect more comprehensively the spectrum of SpA phenotypes seen in daily routine than would be by each cohort alone.

There is no standardized or generally accepted gold standard for imaging studies in axial SpA [25]. MRI cannot be used simultaneously for classification and for evaluating its diagnostic utility for reasons of conceptual circularity [26]. Rheumatologist expert opinion, which was consistently used as gold standard in three SpA classification criteria sets (Amor [27], European Spondylarthropathy Study Group (ESSG) [28], ASAS [1]), served as gold standard for diagnosis of axial SpA. In both inception cohorts, clinician expert opinion by one local rheumatologist (UW for cohort A and WPM for cohort B) based on clinical examination inclusive of standardized evaluation of inflammatory back pain according to Calin criteria [29] and of structured questionnaires on SpA-related features (modified Outcome in Ankylosing Spondylitis International Study (OASIS) protocol [30] for cohort A and Spondyloarthritis Research Consortium of Canada (SPARCC) protocol [31] for cohort B), pelvic radiographs and laboratory values (human leucocyte antigen B27 (HLA-B27), C-reactive protein (CRP)) served to classify the back pain patients as having nr-axSpA (n = 20 and 31 for cohorts A and B, respectively), ankylosing spondylitis (AS; n = 10 and 24 for cohorts A and B, respectively) and nonspecific back pain (NSBP; n = 39 and 33 for cohorts A and B, respectively). Two readers at each site independently categorized pelvic radiographs according to the modified New York criteria [32], and discrepancies were solved by consensus. Current or previous treatment with biologics was an exclusion criterion in both cohorts. The study protocols were approved by the local Ethics Review Boards (Ethics Committee of the University of Alberta, Edmonton, Canada; Kantonale Ethikkommission Zurich, Zurich, Switzerland) and written informed consent was obtained from the study participants.

### Evaluation of magnetic resonance images

The technical parameters for short tau inversion recovery (STIR) and T1 spin echo (T1SE) sequences of semicoronal MRI SIJ scans have been reported previously [17]. The MRI scans (1.5 T Avanto, Siemens Medical Solutions, Erlangen, Germany, for cohorts A and B) were read and scored independently by five blinded readers (one radiologist: VZ; four rheumatologists: SJP, UW, WPM and ZZ). The images of each cohort were evaluated separately in random order on electronic work stations, and MRI scores were entered into a customized online data entry module.

The evaluation of the cartilaginous compartment on SIJ MRI followed a standardized module [16, 17, 23] comprising two sections. The first section represented a global assessment indicating presence/absence of SpA according to both structural and active MRI features by viewing T1SE and STIR sequences simultaneously. The confidence in the global assessment was expressed by a numeric rating scale (NRS) score ranging from 0 (definitely nonSpA) to 10 (definitely SpA). The second section contained a detailed assessment recording BME and fat metaplasia according to standardized lesion definitions and a reference SIJ MRI set developed by consensus amongst study investigators [33]. BME and fat metaplasia in the bone marrow of the cartilaginous joint compartment were recorded as present/absent in each quadrant (upper and lower ilium, upper and lower sacrum) of each SIJ, based on evaluation of each MRI slice [17].

The assessment of the ligamentous compartment on SIJ MRI also comprised a global and a lesion-based section. Readers reported in the global section whether the additional evaluation of the ligamentous joint compartment provided an incremental value (expressed by the three categories “essential”, “contributory” or “noncontributory”) over and above the global assessment based on the cartilaginous joint compartment alone. Readers also recorded whether the additional evaluation of the ligamentous joint compartment changed diagnosis (from nonSpA to SpA or vice versa) or altered the confidence in a diagnosis of SpA based on MRI assessment of the cartilaginous compartment alone. The complex anatomy of the ligamentous joint compartment precluded an evaluation per joint quadrants. BME and fat metaplasia were recorded as present/absent if observed in bone marrow on any slice of the ligamentous compartment. Erosions were not scored in the ligamentous compartment because they cannot be discriminated reliably from physiological insertions of SIJ ligaments.

### Statistical analysis

#### Data description

Differences in demographic and clinical characteristics between cohorts A and B were assessed by Fisher’s exact test for nominal and Wilcoxon test for continuous variables. We calculated the mean percentage of patients and controls over five readers showing ≥1 BME or ≥1 fat metaplasia lesion (dichotomously indicating 'presence' or 'absence' of lesions) in the cartilaginous and ligamentous SIJ compartment, respectively.

#### Interobserver agreement on MRI lesions in both SIJ compartments

Interobserver reliability of MRI lesions was evaluated in both joint compartments because it might be expected that detection of lesions in the ligamentous compartment would be less reliable due to its more complex anatomy. Percent agreement (positive and negative concordance) among the 10 possible reader pairs for each group of study subjects, and weighted Cohen’s kappa values for five readers jointly per cohort [34] were used to calculate the reproducibility of BME and fat metaplasia among the five readers for the cartilaginous and ligamentous SIJ compartments. Kappa values were not calculable for all categories of study subjects due to small sample sizes of n = 10–39 for nr-axSpA, AS, NSBP and healthy controls (HC) [35]. The inter-reader agreement was defined as slight, fair, moderate, substantial and almost perfect by values of the weighted Cohen’s kappa κ < 0.2, κ = 0.2– < 0.4, κ = 0.4– < 0.6, κ = 0.6– < 0.8, and κ = 0.8–1, respectively [36]. For kappa values, we provide bootstrap confidence intervals based on 1000 bootstrap replications and computed at a confidence level of 95 %.

#### MRI lesions in the cartilaginous versus ligamentous joint compartment

We calculated the mean percentage of patients and controls over five readers who showed ≥1 MRI lesion both in the cartilaginous and ligamentous compartment, and in only one or in none of the two joint compartments, separately for BME and fat infiltration. The constellation of lesions observed solely in the ligamentous compartment without corresponding lesions in the cartilaginous compartment was further analyzed regarding agreement among the five readers.

#### Impact of the ligamentous joint compartment on classification

We calculated the mean percentage of patients and controls over five readers where the additional evaluation of the ligamentous compartment was regarded as essential, contributory or noncontributory for diagnosis of SpA versus nonSpA beyond the assessment of the cartilaginous compartment alone. We further analyzed whether the additional evaluation of the ligamentous compartment led to a switch in diagnosis (assessment of the cartilaginous compartment alone indicating nonSpA that changes to SpA by additional evaluation of the ligamentous compartment). We also assessed how often confidence in diagnosis of SpA increased from 5–7 to 8–10 following additional evaluation of the ligamentous compartment, and we expressed this as mean percentage of patients and controls over five readers.

We used R Core Team 2014, version 3.1.0, Vienna, Austria, for statistical analysis [37].

## Results

### Data description

Table 1 displays the characteristics of the two SpA inception cohorts representing a wide spectrum of SpA phenotypes. The two strategies to recruit patients with early SpA resulted in substantial demographical and clinical differences between the two cohorts, with cohort B having longer symptom duration and less disease activity. This disparity with regards to symptom duration, age of study participants and disease activity justified analysis of the two cohorts separately.

**Table 1:** Demographical and clinical characteristics of two spondyloarthritis inception cohorts, and percentage of subjects with bone marrow edema or fat metaplasia in the cartilaginous and ligamentous compartments of the sacroiliac joints (mean percentage over five readers)

Cohort	A (n = 89)				B (n = 88)		
Recruitment mode	Suspected axial SpA by practising rheumatologists or PCP				AAU plus past or present back pain		
Group	nr-axSpA	AS	NSBP	HC	nr-axSpA	AS	NSBP
Number of subjects	20	10	39	20	31	24	33
Male:female (% male)	11:9 (55.0)	8:2 (80.0)	11:28 (28.2)	7:13 (35.0)	17:14 (54.8)	11:13 (45.8)	17:16 (51.5)
Age (years)	32.2 (12.3)	30.0 (9.5)*	32.7 (11.5)	30.6 (6.5)	36.2 (12.1)	41.5 (7.1)*	33.6 (15.7)
Symptom duration (years)	1.3 (1.8)*	3.9 (1.8)*	NA	NA	10.0 (14.0)*	12.5 (13.5)*	NA
HLA B27 positive (%)	12 (60.0)	9 (90.0)	ND	ND	24 (80.0)^b^	21 (87.5)	10 (55.6)^b^
BASDAI (NRS)	4.4 (3.1)^a^	5.4 (1.5)^a^	NA	NA	3.5 (4.4)	2.0 (3.4)^b^	NA
BASFI (NRS)	1.8 (3.9)*^a^	2.7 (1.5)^a^	NA	NA	0.8 (2.3)*	0.6 (2.8)^b^	NA
CRP (mg/l)	4.0 (4.5)^a^	5.0 (8.0)^a^	ND	ND	2.7 (5.2)	8.0 (8.7)^b^	0.9 (0.9)^b^
BME cart	83.0	88.0	27.7	34.0	45.2	74.2	27.9
BME lig	42.0	80.0	3.6	6.0	11.6	38.3	2.4
Fat cart	47.0	66.0	29.2	18.0	43.9	79.2	22.4
Fat lig	21.0	46.0	17.9	12.0	25.2	42.5	9.1

Values for patient characteristics are the median (interquartile range; expressed as difference between first and third quartile) unless otherwise stated

*P ≤ 0.05 (Fisher’s exact test) between cohorts A and B

^a^Cohort A: CRP values are based on 18, and BASDAI/BASFI values on 19 patients with nr-axSpA, respectively; BASDAI/BASFI and CRP values are based on 9 patients with AS

^b^Cohort B: HLA B27 values are based on 30 patients with nr-axSpA; BASDAI/BASFI and CRP values are based on 22 patients with AS; in NSBP patients, HLA B27 and CRP values are based on 18 subjects; the prevalence of HLA B27 in patients with AAU in general is 50–60 % [41]

*AAU* Acute anterior uveitis, *AS* Ankylosing spondylitis, *BASDAI* Bath Ankylosing Spondylitis Disease Activity Index, *BASFI* Bath Ankylosing Spondylitis Functional Index, *BME* Bone marrow edema, *Cart* Cartilaginous compartment of the sacroiliac joint, *CRP* C-reactive protein (reference range ≤5 mg/l), *Fat* Fat metaplasia, *HC* Healthy control, *HLA B27* Human leucocyte antigen B27, *Lig* Ligamentous compartment of the sacroiliac joint, *NA* Not applicable, *ND* Not done, *nr-axSpA* Nonradiographic axial spondyloarthritis, *NRS* Numeric rating scale, *NSBP* Nonspecific back pain, *PCP* Primary care physician

MRI bone marrow lesions were less frequent in the ligamentous than cartilaginous compartment, consistently across both cohorts, all study groups, and for both lesion types. Among nr-axSpA patients, BME in the ligamentous/cartilaginous compartment occurred in 42.0/83.0 % and 11.6/45.2 % for cohorts A and B, respectively, and fat metaplasia in 21.0/47.0 % and 25.2/43.9 %, respectively (Table 1). The same disparity was observed in AS patients, but with higher overall lesion frequencies. The disparity in MRI lesion frequency between the cartilaginous and the ligamentous compartment was particularly pronounced for BME in NSBP patients. In cohorts A and B, 27.7 and 27.9 % of NSBP patients had BME in the cartilaginous versus only 3.6 and 2.4 % in the ligamentous compartment, respectively.

In cohorts A and B, BME in the ligamentous compartment was recorded in 80.0 and 38.3 % of AS patients, respectively, compared to 42.0 and 11.6 % of nr-axSpA patients. Similarly, fat metaplasia in the ligamentous compartment was observed more often in AS (46.0 and 42.5 % for cohorts A and B, respectively) than in nr-ax-SpA (21.0 and 25.2 %, respectively) patients. However, a comparable difference in lesion frequency between AS and nr-axSpA groups was also recorded for the cartilaginous compartment. BME in the cartilaginous compartment was reported in 88.0 and 74.2 % of AS and 83.0 and 45.2 % of nr-axSpA patients in cohorts A and B, and fat metaplasia in 66.0 and 79.2 % of AS and 47.0 and 43.9 % of nr-axSpA patients.

### Interobserver agreement on MRI lesions in both joint compartments

Agreement by percent concordance among 10 possible reader pairs for BME and fat metaplasia showed no systematic discrepancies between the two joint compartments across both cohorts (Table 2). In cohort A, Cohen’s kappa over five readers jointly for BME was substantial and comparable for the cartilaginous (kappa = 0.66) and ligamentous (kappa = 0.68) compartment. In cohort B, kappa for BME was substantial in the cartilaginous compartment (0.64), but fair for the ligamentous compartment (0.37). In cohorts A and B, Cohen’s kappa for fat metaplasia was moderate in the cartilaginous compartment (0.40/0.51 in cohorts A and B, respectively) and fair for the ligamentous compartment (0.36/0.39 in cohorts A and B, respectively).

**Table 2:** Agreement as percent concordance among 10 possible reader pairs for each group of study subjects (kappa values for five readers jointly per cohort)

Cohort	A (n=89)				B (n=88)		
Group	nr-axSpA	AS	NSBP	HC	nr-axSpA	AS	NSBP
Number of subjects	20	10	39	20	31	24	33
Percent concordance among 10 reader pairs^a^							
Total (positive/negative)							
BME cart	88.0 (77.0/11.0)	88.0 (82.0/6.0)	78.5 (16.9/61.5)	85.0 (26.5/58.5)	78.1 (34.2/43.9)	78.3 (63.3/15.0)	86.7 (20.6/66.1)
BME lig	78.0 (31.0/47.0)	92.0 (76.0/16.0)	93.8 (0.5/93.3)	89.0 (0.5/88.5)	87.7 (5.5/82.3)	61.7 (19.2/42.5)	97.0 (0.9/96.1)
Fat cart	69.0 (31.5/37.5)	64.0 (48.0/16.0)	74.9 (16.7/58.2)	74.0 (5.0/69.0)	76.8 (32.3/44.5)	70.8 (64.6/6.3)	76.4 (10.6/65.8)
Fat lig	77.0 (9.5/67.5)	64.0 (28.0/36.0)	82.1 (9.0/73.1)	86.0 (5.0/81.0)	77.4 (13.9/63.5)	60.8 (22.9/37.9)	89.1 (3.6/85.5)
Kappa value for five readers jointly (95 % CI)^b^							
BME cart	0.66 (0.56–0.75)				0.64 (0.53–0.72)		
BME lig	0.68 (0.55–-0.79)				0.37 (0.26–0.45)		
Fat cart	0.40 (0.32–0.49)				0.51 (0.42–0.60)		
Fat lig	0.36 (0.25–0.45)				0.39 (0.25–0.51)		

^a^Percent concordance among 10 possible reader pairs (100 % = 10 concordant reader pairs); Total = positive and negative concordance for presence/absence of BME or fat metaplasia in the cartilaginous and ligamentous compartment of the sacroiliac joints

^b^Weighted Cohen’s kappa values for all five readers jointly

*AS* Ankylosing spondylitis, *BME* Bone marrow edema, *Cart* Cartilaginous compartment of the sacroiliac joint, *CI* 95 % confidence interval, *Fat* Fat metaplasia, *HC* Healthy control, *Lig* Ligamentous compartment of the sacroiliac joint, *nr-axSpA* Nonradiographic axial spondyloarthritis, *NSBP* Nonspecific back pain, *R* Reader, *RP* Reader pair

### MRI lesions in the cartilaginous versus ligamentous joint compartment

MRI lesions occurring concomitantly in the cartilaginous and ligamentous SIJ compartment were recorded more frequently in AS than in nr-axSpA patients, both for BME and fat metaplasia and across both cohorts A and B (BME: 80.0 and 37.5 % in AS, and 42.0 and 11.6 % in nr-axSpA; fat metaplasia: 42.0 and 41.7 % in AS, and 21.0 and 25.2 % in nr-axSpA, respectively) (Table 3). By contrast, concomitant BME in both joint compartments was consistently very rare in controls of both cohorts A and B (NSBP patients 2.1 and 2.4 %; healthy controls in cohort A 4.0 %). Thus, the constellation of concomitant BME lesions in both the cartilaginous and ligamentous compartment showed more frequently in nr-axSpA (42.0 and 11.6 %) than in NSBP patients (2.1 and 2.4 %) in cohorts A and B. BME or fat metaplasia lesions solely in the ligamentous compartment were virtually absent for all groups of study subjects across both cohorts (0–2.0 % for BME and 0–4.0 % for fat metaplasia, respectively), and they were never recorded at the same site by more than one out of five readers.

**Table 3:** Bone marrow edema and fat metaplasia in the cartilaginous versus ligamentous sacroiliac joint compartment (mean percentage of subjects over five readers)

Cohort	A (n=89)				B (n=88)		
Group	nr-axSpA	AS	NSBP	HC	nr-axSpA	AS	NSBP
Number of subjects	20	10	39	20	31	24	33
BME cart+/lig+	42.0	80.0	2.1	4.0	11.6	37.5	2.4
BME cart+/lig–	41.0	8.0	25.6	30.0	33.5	36.7	25.5
BME cart–/lig+	0	0	1.5^a^	2.0^a^	0	0.8^a^	0
BME cart–/lig–	17.0	12.0	70.8	64.0	54.8	25.0	72.1
Fat cart+/lig+	21.0	42.0	17.4	12.0	25.2	41.7	8.5
Fat cart+/lig–	26.0	24.0	11.8	6.0	18.7	37.5	13.9
Fat cart–/lig+	0	4.0^b^	0.5^b^	0	0	0.8^b^	0.6^b^
Fat cart–/lig–	53.0	30.0	70.3	82.0	56.1	20.0	77.0

^a^BME cart–/lig+: three NSBP patients and two HC by one single reader in cohort A; one AS patient by one reader in cohort B

^b^Fat cart–/lig+: two AS and one NSBP patient by three different readers in cohort A; one AS and one NSBP patient by two different readers in cohort B

*AS* Ankylosing spondylitis, *BME* Bone marrow edema, *Cart* Cartilaginous compartment of the sacroiliac joint, *Fat* Fat metaplasia, *HC* Healthy control, *Lig* Ligamentous compartment of the sacroiliac joint, *nr-axSpA* Nonradiographic axial spondyloarthritis, *NSBP* Nonspecific back pain

### Impact of the ligamentous joint compartment on diagnosis

The additional evaluation of the ligamentous compartment for diagnosis of SpA versus nonSpA over and above the assessment of the cartilaginous compartment alone was regarded as essential by one single reader in one nr-axSpA patient of cohort B enhancing the confidence in a diagnosis of SpA from 7 to 10 on a NRS ranging from 0–10 (Table 4). Otherwise none of the ligamentous joint assessments in any of the two cohorts were reported to be essential by the five readers. In only 28.0 and 7.7 % of nr-axSpA patients in cohorts A and B, respectively, was the evaluation of the ligamentous compartment considered contributory to diagnosis. The additional assessment of the ligamentous compartment was regarded as noncontributory for diagnosis in 72.0 and 91.6 % of nr-axSpA patients in cohorts A and B. None of the ligamentous joint assessments led to a change in diagnosis.

**Table 4:** Impact of assessment of the ligamentous sacroiliac joint compartments on diagnosis of SpA versus nonSpA (mean percentage of subjects over five readers)

Cohort	A (n=89)				B (n=88)		
Group	nr-axSpA	AS	NSBP	HC	nr-axSpA	AS	NSBP
Number of subjects	20	10	39	20	31	24	33
Lig essential for diagnosis of SpA vs Non-SpA	0	0	0	0	0.6^c^	0	0
Lig contributory for diagnosis of SpA vs Non-SpA	28.0	64.0	2.1	1.0	7.7	26.7	0
Lig non-contributory for diagnosis of SpA vs Non-SpA	72.0	36.0	97.9	99.0	91.6	73.3	100
Lig inducing change in diagnosis^a^	0	0	0	0	0	0	0
Lig inducing increase in confidence in diagnosis^b^	0	0	0	0	0.6^c^	0	0

^a^Change in diagnosis: assessment of the cartilaginous compartment alone indicating nonSpA (confidence 0–5) changed to SpA (confidence 5–10) by additional evaluation of the ligamentous compartment

^b^Increase in confidence of diagnosis: confidence in diagnosis of SpA increased from 5–7 to 8–10 following additional evaluation of the ligamentous compartment

^c^One nr-axSpA patient by one reader in cohort B

*AS* Ankylosing spondylitis, *HC* Healthy control, *Lig* Ligamentous compartment of the sacroiliac joint, *nr-axSpA* Nonradiographic axial spondyloarthritis, *NSBP* Nonspecific back pain, *SpA* Spondyloarthritis

## Discussion

**Fig. 2:**
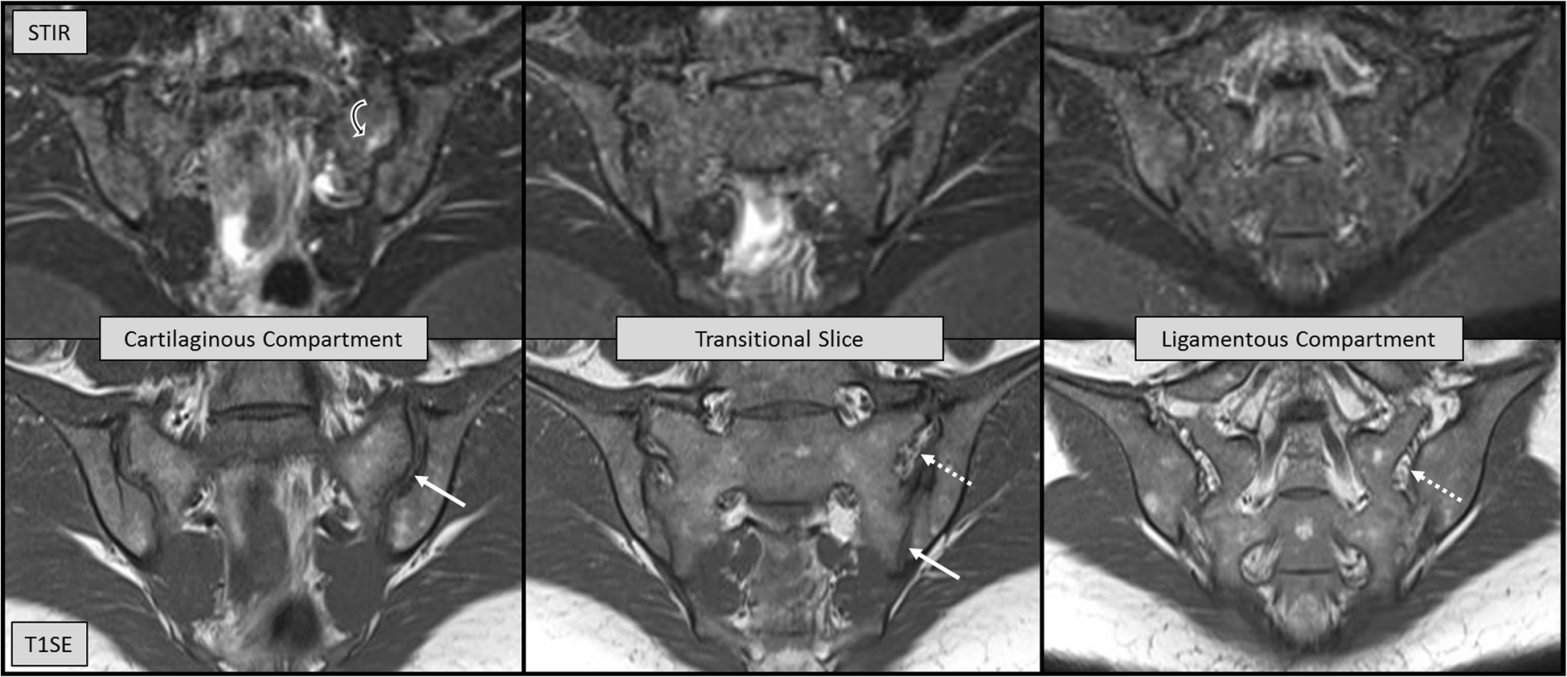
Thirty-one-year-old woman with remitting mechanical back pain over several years. The sacral part of the left sacroiliac joint in the cartilaginous compartment demonstrates increased signal intensity compatible with bone marrow edema (*curved white and black arrows* on STIR), which is not visible on the transitional slice nor in the ligamentous compartment. See legend to Fig. 1 for details on the structure of the figure. *STIR* short tau inversion recovery, *T1SE* T1 spin echo

In two SpA inception cohorts reflecting routine practice with broad variation of symptom duration and disease activity, assessment of the ligamentous compartment on SIJ MRI provided no incremental value for diagnosis of axial SpA compared to the traditional evaluation of the cartilaginous SIJ compartment alone. Redundancy of MRI lesions in the ligamentous and cartilaginous compartment is the most likely explanation: nearly all SpA and control subjects having BME and/or fat metaplasia in the bone marrow of the ligamentous SIJ compartment showed the same lesion also in the cartilaginous compartment. However, concomitant BME in the cartilaginous and ligamentous compartments was observed more frequently in nr-axSpA than in NSBP patients (sensitivity/specificity 0.42/0.98 and 0.12/0.98 for cohorts A and B, respectively), which may assist towards discrimination of the two conditions (Figs. 1 and 2).

Lesions in the ligamentous SIJ compartment were more frequently reported in AS than in nr-axSpA patients. This observation is in agreement with the findings of an earlier retrospective study, which reported BME in enthesial SIJ structures more commonly in AS (40–86 %) than in patients with early undifferentiated SpA (26–43 %) [10]. A study recruiting 37 patients who fulfilled the ESSG criteria showed a statistically significant association of ligamentous SIJ inflammation with AS according to the modified New York criteria [21]. These earlier data suggested that inflammation in the ligamentous SIJ compartment may be indicative of longstanding rather than early disease. However, our findings question this hypothesis as we observed a comparable difference in lesion frequency between nr-axSpA and AS groups also in the cartilaginous compartment suggesting an overall accumulation of inflammatory lesions in both compartments over time. Only a follow-up study assessing both SIJ compartments longitudinally has the potential to clarify this issue.

Agreement for BME was substantial both in the cartilaginous and ligamentous compartment (kappa for five readers jointly 0.66 and 0.68, respectively) in cohort A. In cohort B, agreement was substantial in the cartilaginous compartment (kappa 0.64) and fair in the ligamentous compartment (kappa 0.37), the latter finding possibly attributable to a much lower frequency of ligamentous BME lesions compared to cohort A. A Dutch study on SIJ MRI in 68 patients with inflammatory back pain, but lacking a control group, showed comparable agreement on BME in the cartilaginous compartment (kappa for two readers for right/left SIJ 0.73/0.65), but a lower agreement in the ligamentous compartment (kappa for right/left SIJ 0.49/0.38) [38]. In our study, agreement for fat metaplasia in both joint compartments was lower than for BME.

An additional assessment of the ligamentous SIJ compartment provided no incremental value for diagnosis of axial SpA nor for confidence with this diagnosis over and above the traditional approach, in which only the cartilaginous compartment of the SIJ is evaluated. This finding is most likely due to a nearly complete redundancy between ligamentous and cartilaginous SIJ MRI lesions. There was virtually no isolated inflammation in the ligamentous SIJ compartment alone without associated lesions in the cartilaginous compartment. This observation is in line with the results of a previous report in which only 1 of 37 patients fulfilling the ESSG criteria showed isolated inflammation of just the ligamentous SIJ compartment [21]. However, in our study, concomitant BME lesions both in the cartilaginous and ligamentous compartment showed more frequently in nr-axSpA (12–42 %) than in NSBP patients (2 %). If confirmed by other studies, this MRI feature may facilitate the clinically often challenging discrimination between the two conditions.

A study limitation shared with most other evaluation modules of SIJ MRI is the lack of additional semi-axial slices, as proposed by the Danish working group [3, 20–22, 39]. Semi-axial slices running perpendicularly to the long axis of the SIJ, in contrast to the traditional semi-coronal imaging of the SIJ, may permit a more precise anatomical localization of MRI lesions by simultaneous visualization of both compartments and their anatomical border on the same image [3, 20]. Both sequences viewed together facilitate the identification of normal MRI features and variants that may mimic SpA-associated inflammatory SIJ lesions on the semi-coronal plane alone [3], such as normal vascular structures mimicking BME or the normal variant of a bipartite ilium suggesting erosion by semi-coronal slices only [2]. An additional semi-axial plane may also help to recognize partial volume artifacts between the cartilaginous and the ligamentous compartment, which may be misinterpreted as erosion on semi-coronal images alone [40].

However, data are lacking whether additional semi-axial slices facilitate evaluation of the ligamentous compartment leading to enhanced diagnostic utility of SIJ MRI in nr-axSpA. Two of our findings, that question a potentially relevant role of additional semi-axial slices, are the low frequency of ligamentous SIJ inflammation in early disease, and the very high association of inflammatory features in both compartments. Another challenge is the technically difficult assessment of the ligamentous compartment. Its irregular contour prevents a quadrant-based evaluation similar to the cartilaginous compartment, and defining a reproducible demarcation where the ligamentous compartment ends posteriorly is challenging.

## Conclusion

Assessment of MRI bone marrow lesions in the ligamentous SIJ compartment provided no incremental value towards diagnosis of nr-axSpA. Moreover, it did not enhance confidence in the diagnosis over and above the traditional evaluation of the cartilaginous compartment alone, when using semi-coronal images. The most likely explanation was a high level of redundancy with MRI BME and fat metaplasia lesions occurring concomitantly in the ligamentous and cartilaginous compartments. However, BME observed simultaneously in both SIJ compartments might facilitate the clinically relevant discrimination between nr-axSpA and NSBP patients because the latter showed concomitant inflammation in both compartments only very rarely.

## Abbreviations

AAU: Acute anterior uveitis
AS: Ankylosing spondylitis
ASAS: Assessment of SpondyloArthritis International Society
BME: Bone marrow edema
CRP: C-reactive protein
ESSG: European Spondylarthropathy Study Group
HC: Healthy controls
HLA B27: Human leucocyte antigen B27
MRI: Magnetic resonance imaging
nr-axSpA: Non-radiographic axial spondyloarthritis
NRS: Numeric rating scale
NSBP: Nonspecific back pain
OASIS: Outcome in Ankylosing Spondylitis International Study
SIJ: Sacroiliac joints
SpA: Spondyloarthritis
SPARCC: Spondyloarthritis Research Consortium of Canada
STIR: Short tau inversion recovery
T1SE: T1 spin echo
